# Remote Patient Monitoring Technologies for Predicting Chronic Obstructive Pulmonary Disease Exacerbations: Review and Comparison

**DOI:** 10.2196/16147

**Published:** 2020-05-21

**Authors:** Kathleen G Fan, Jess Mandel, Parag Agnihotri, Ming Tai-Seale

**Affiliations:** 1 New York Medical College Valhalla, CA United States; 2 Division of Pulmonary, Critical Care, and Sleep Medicine School of Medicine University of California San Diego La Jolla, CA United States; 3 University of California San Diego Health Population Health Services Organization University of California San Diego La Jolla, CA United States; 4 Department of Family Medicine and Public Health University of California San Diego La Jolla, CA United States

**Keywords:** COPD, disease exacerbation, remote patient monitoring, mobile health, telehealth, at-home monitoring, remote monitoring system, wearable

## Abstract

**Background:**

Chronic obstructive pulmonary disease (COPD) is the third leading cause of death by disease worldwide and has a 30-day readmission rate of 22.6%. In 2015, COPD was added to the Medicare Hospital Readmission Reductions Program.

**Objective:**

The objective of this paper was to survey the current medical technologies for remote patient monitoring (RPM) tools that forecast COPD exacerbations in order to reduce COPD readmissions.

**Methods:**

We searched literature and digital health news to find commercially available RPM devices focused on predicting COPD exacerbations. These technologies were reviewed and compared according to four criteria: forecasting ability, cost, ease of use, and appearance. A rating system was developed to facilitate the evaluation process.

**Results:**

As of June 2019, a list of handheld and hands-free devices was compiled. We compared features and found substantial variations. Devices that ranked higher on all criteria tended to have a high or unlisted price. Commonly mass-marketed devices like the pulse oximeter and spirometer surprisingly fulfilled the least criteria.

**Conclusions:**

The COPD RPM technologies with most technological promise and compatibility with daily living appear to have high or unlisted prices. Consumers and providers need better access to product information to make informed decisions.

## Introduction

Chronic obstructive pulmonary disease (COPD) is the third leading cause of death worldwide and has a 30-day hospital readmission rate of 22.6% [1,2]. Its exacerbations, if not well managed, can reduce patients’ quality of life and increase costs of care [3-6]; COPD is a target condition in the Medicare Hospital Readmission Reductions Program.

Early COPD exacerbation recognition has been a focus of inquiries since faster intervention correlates with better outcomes [7-9]. Oxygen saturation, respiratory rate (RR), and heart rate (HR) have been identified as useful biomarkers [10-14]. Measuring oxygen saturation alone, however, may not be sufficient because it naturally fluctuates throughout the day [14,15]. Measuring these biomarkers in the form of remote patient monitoring (RPM) technologies has improved patients’ abilities to self-manage and decreases COPD’s economic and clinical burden [9,16,17].

This paper reviews RPM technologies targeting early COPD exacerbation markers. We provide a concise, practical comparison of technologies to assist consumers and providers in making informed decisions about available technologies.

## Methods

We examined available and commercialized devices by searching for COPD RPM technologies in the literature. We searched Medline and ClinicalTrials.gov and digital health news outlets such as MobiHealthNews, Wearable Technologies, and FierceBiotech with keywords “COPD,” “COPD exacerbation,” “AECOPD,” and “chronic obstructive pulmonary disease” in combinations with “remote patient monitoring,” “at-home monitoring,” “remote monitoring system,” “device,” “wearable,” and “technology” in June 2019. We narrowed the focus to FDA-approved devices or ones slated for approval within a year. We grouped selected technologies into handheld versus hands-free devices and compared them using four additional criteria: forecasting ability, cost, ease of use, and appearance. Forecasting ability is the likelihood the device would signal a developing COPD exacerbation based on indicative biomarkers [10-14]. Costs were compared at retail prices, if listed. Ease of use was assessed based on efforts required from users to operate the device because patients are more likely to consistently use passive and user-friendly devices [18-20]. Appearance was considered because it influences user acceptability. Unattractive or uncomfortable devices result in lower take-up [18-20].

Using a star scheme, we rated each device for all criteria besides cost where actual amount (if available) was considered. One star indicates poor fulfillment, three stars indicates adequate fulfillment, and five stars indicates excellent fulfillment of that criterion (Table 1).

**Table 1:** Comparison of selected chronic obstructive pulmonary disease handheld and hands-free remote monitors.

Device		Forecasting ability	Cost	Ease of use	Appearance
**Handheld**					
	Spirometer	★	$99-$2500	★	★★★
	Pulse oximeter	★	$15-$599	★	★
	Propeller Health sensor	★	unlisted	★★★	★★★
	Cohero Health kit	★★★	$49/mo	★★★	★★★
**Hands-free**					
	Spry Health Loop System	★★★★★	unlisted	★★★★★	★★★★★
	Omron HeartGuide	★★★	$499	★★★★★	★★★★★
	Spire Health Tag	★★★★★	$49	★★★	★★★
	Cosinuss One	★	$146.50	★★★	★★★
	Current Health Armband	★★★★★	$199 + $40/mo	★★★	★
	Adamm RSM	★★★★★	unlisted	★★★★★	★★★★★

## Results

### Handheld Monitors

Spirometers are the gold standard pulmonary function test for COPD diagnosis. With the rise of over-the-counter tabletop to handheld spirometers, at-home spirometry has become common for daily monitoring of the amount and/or speed of air that can be inhaled and exhaled. Spirometers range in cost from a few hundred to several thousand dollars [21,22].

Pulse oximeters are lightweight devices measuring oxygen saturation that have been mass-marketed for at-home care. Pulse oximeters can cost from $15 to $599 [23,24].

The Propeller Health sensor is an electronic inhaler attachment tracking medication use and potential exacerbations (≥10 inhaler puffs within a 24-hour period or greater usage over a 48-hour period) [25,26]. A study showed 17 of a 39-patient cohort adhered to using the device over a 2-year study [27]. The price of Propeller’s asthma sensor is negotiated with health care delivery organizations or payers. It can be free to patients through sponsored health plans but is otherwise around $300 [28]. The price of its COPD sensor is not listed.

The Cohero Health kit includes a medical-grade handheld spirometer, medication-tracking sensor, and web app that centralizes the data [29,30]. The kit comes as a subscription service for $49 per month [31].

### Hands-Free Monitors

Spry Health’s Loop System is a wristband monitor. It tracks oxygen saturation, HR, RR, and blood pressure, alerting on significant changes in the wearer’s physiological data [32]. Its price is unlisted.

Omron’s HeartGuide is a watch-like monitor measuring HR, blood pressure, physical activity, and sleep quality. These features are transmitted to a mobile app where patients can track progress and access health coaching [33]. Although the HeartGuide directly targets patients with heart conditions, its ability to track HR, physical activity, and sleep quality can help detect signs of COPD exacerbation [34,35]. The HeartGuide costs $499.

The Spire Health Tag is a disposable adhesive sensor that attaches to clothing. Each tag tracks RR, HR, breathing pattern, sleep quality, and physical activity, which are logged into a smartphone app [36]. The tags have a 1-year battery life and are washer and dryer safe. Each tag costs $49. Consumers can opt for a Spire membership, which at $10 per month provides free replacements and additional tags for $25 each [37].

The Cosinuss One is a monitor placed in the ear that connects to a smartphone app to measure HR, HR variability, and body temperature. Cosinuss is currently developing OxMotion, an add-on specific to COPD patients tracking RR and oxygen saturation levels. We primarily discuss Cosinuss One because the OxMotion is not yet available. The Cosinuss One costs $146.50 [38].

Current Health’s Remotely Monitor system is an armband monitor measuring HR, RR, skin temperature, oxygen saturation, and movement. Data are transmitted to a cloud platform and can be integrated into the patient’s electronic health record. At the most basic membership, the Current Armband is $199 upfront and $40 per month for continuous service [39].

The Adamm RSM from Health Care Originals is an adhesive device for the upper torso that monitors cough rate, respiratory patterns, HR, and temperature. Data go to a mobile app and web portal viewable by patients and their physicians [40]. Its price is unlisted.

## Discussion

### Principal Findings

Selected COPD RPM devices were assessed based on forecasting ability, cost, ease of use, and appearance. Spry Health Loop System and Adamm RSM ranked highest across most dimensions aside from cost. The pulse oximeter fulfilled the least criteria.

The Loop System, Current Health Armband, Spire Health Tag, and Adamm RSM ranked highest in forecasting ability since they monitor the most indicative biomarkers. Although Cosinuss One tracks multiple biomarkers, it has shown inaccurate measurements in external studies and thus may not have high forecasting ability [41-43]. Adamm RSM and Spire Health Tag track respiratory patterns, which may be even more accurate [12].

Spirometers, although a well-established COPD diagnostic method, ranked low in forecasting ability. Spirometry, when conducted in outpatient settings or unaided by a health care professional, can often yield inaccurate results due to technical factors [7,44]. Cohero Health’s mobile spirometer is International Organization for Standardization (ISO) 9001 and 13485 certified, giving it more accuracy than other mass-marketed spirometers [30]. Although the Propeller sensor is easy to use and has been used to predict incoming exacerbations when it detects increased use of the inhaler, it does not provide data on pulmonary function considered important to predict an incoming exacerbation [27]. Both Cohero and Propeller require patients to use the inhaler, which could face challenges in adherence [18,26].

The Loop System and HeartGuide ranked highest in ease of use. Both devices are wristbands that can be worn without effortful engagement from users to obtain data, which increases usability and adherence [45]. Adamm RSM similarly can be easily hidden under shirts. Other devices with three stars required a little more adjusting. Current Health Armband and pulse oximeter are easy to wear but may shift throughout the day due to their locations on the arm and finger, respectively.

Although relatively easy to use, the Current Health Armband ranked lowest in appearance due to its bulky design and high-profile arm placement. Pulse oximeters also ranked low due to high visibility. While patients can choose to spot check rather than continuously wear the device, this would require some reminder, therefore decreasing its ease of use.

Other devices were relatively low profile. The Loop and HeartGuide are modeled after watches while the handheld spirometer and Propeller inhaler can be carried in a pocket or bag. The Spire Health Tag is out of view once attached to clothing. Adherence becomes challenged if consumers change or discard clothing with the Health Tag attached, however. The Cosinuss One is a low-profile earpiece but may be disadvantageous because it may impede hearing.

Cost-wise, the Spire Health Tag seemingly ranks lowest. But because the sensor may only be attached once, users must decide which clothing they wear most frequently and wear those items every day. Over-the-counter options such as spirometers and pulse oximeters, although inexpensive compared to other RPM devices, are often inaccurate in measurement and have less robust premarket testing [7,46].

### Limitations

This review is not exhaustive. It provides a review of devices currently on the market and readily searchable online. Some technologies were not selected because they were similar in function but not yet on the market or lacked product information. Future efforts are necessary to update this review, given digital health technology is continually improving and evidence for efficacy of RPM in reducing COPD exacerbations is still developing [47]. Another limitation concerns the star rating system, which is based on qualitative assessments rather than quantitative metrics [47]. Last, technology is only one aspect of COPD management strategy. More guideline-concordant treatment and better patient engagement are needed, but they are beyond the scope of this brief report [48].

### Conclusion

Patients can better manage their COPD with the aid of RPM technology that can be easily adopted into their daily routine. The most promising devices are either expensive or without available cost information. Consumers and health care organizations can benefit from more publicly accessible information on COPD RPM products and their comparative effectiveness and costs.

## Abbreviations

COPD: chronic obstructive pulmonary disease
HR: heart rate
ISO: International Organization for Standardization
RPM: remote patient monitoring
RR: respiratory rate
